# PD Control Compensation Based on a Cascade Neural Network Applied to a Robot Manipulator

**DOI:** 10.3389/fnbot.2020.577749

**Published:** 2020-12-03

**Authors:** Luis Arturo Soriano, Erik Zamora, J. M. Vazquez-Nicolas, Gerardo Hernández, José Antonio Barraza Madrigal, David Balderas

**Affiliations:** ^1^Departamento de Ingeniería Mecánica Agrícola, Universidad Autónoma Chapingo, Texcoco, Mexico; ^2^Laboratorio de Robótica y Mecatrónica, Instituto Politécnico Nacional, CIC, Ciudad de México, Mexico; ^3^Unidad Mixta Internacional, French-Mexican Laboratory of Informatics and Automatic Control, 3175 French National Research Council, Centro de Investigación y de Estudios Avanzados del Instituto Politécnico Nacional, Deparment of Control of Dynamic Systems, Ciudad de México, Mexico; ^4^Unidad Profesional Adolfo López Mateos, Escuela Superior de Ingeniería Química e Industrias Extractivas del Instituto Politécnico Nacional, Academia de Física, Ciudad de México, Mexico; ^5^Tecnologico de Monterrey, Escuela de Ingeniería y Ciencias, Ciudad de México, Mexico

**Keywords:** cascade neural networks, robot manipulator, PD control, radial basis function, control compensation

## Abstract

A Proportional Integral Derivative (PID) controller is commonly used to carry out tasks like position tracking in the industrial robot manipulator controller; however, over time, the PID integral gain generates degradation within the controller, which then produces reduced stability and bandwidth. A proportional derivative (PD) controller has been proposed to deal with the increase in integral gain but is limited if gravity is not compensated for. In practice, the dynamic system non-linearities frequently are unknown or hard to obtain. Adaptive controllers are online schemes that are used to deal with systems that present non-linear and uncertainties dynamics. Adaptive controller use measured data of system trajectory in order to learn and compensate the uncertainties and external disturbances. However, these techniques can adopt more efficient learning methods in order to improve their performance. In this work, a nominal control law is used to achieve a sub-optimal performance, and a scheme based on a cascade neural network is implemented to act as a non-linear compensation whose task is to improve upon the performance of the nominal controller. The main contributions of this work are neural compensation based on a cascade neural networks and the function to update the weights of neural network used. The algorithm is implemented using radial basis function neural networks and a recompense function that leads longer traces for an identification problem. A two-degree-of-freedom robot manipulator is proposed to validate the proposed scheme and compare it with conventional PD control compensation.

## 1. Introduction

An industrial robot manipulator frequently works at high velocities to reach its desired position. Common tasks performed by robot manipulators include trajectory tracking, reaching positions, and picking and dropping objects. These tasks need the robot controllers to maintain satisfactory dynamic behavior in spite of possible external perturbations, unknown dynamic parameters, and sensor information loss (Armendariz et al., 2014). Several controllers that are often implemented to manage these features are also mentioned (Luo and Kuo, 2016; Makarov et al., 2016; Nicolis et al., 2016; Pan et al., 2018; Hwang and Yu, 2020). Over time, the Proportional Integral Derivative (PID) control has been used to design industrial robots due to their simple structure and simple hardware implementation. However, during operation, the PID integral gain provokes the controller to reduce its bandwidth and stability (Rahimi Nohooji, 2020). PD control with uncertainty compensation has been proposed to manage the increase of integral gain due to the steady-state error. The PD controllers are also limited without gravity compensation, which requires a dynamic model (Wen Yu and Rosen, 2013). In practice, the non-linearities of a dynamic robot system are generally unknown.

To solve this issue, different approaches have been developed in order to compensate for unmodeled uncertainties (i.e., noise, gravity, and friction). Intelligent compensation is a model free and it has been applied to well-known algorithms such as the neural networks (NNs) and fuzzy logic (FL) (Krishna and Vasu, 2018; Wang et al., 2019b). In Liu et al. (2020), the authors propose an adaptive NN backstepping control design for fractional-order non-linear systems with actuator faults whose parameters and patterns are fully unknown. Baek et al. (2016) present an adaptive sliding mode control scheme that implements the time-delay estimation. In Xu et al. (2018), a fuzzy NN sliding mode control is designed to improve controller performance against system uncertainty and external disturbances. Kumar et al. (2012) proposed a hybrid trajectory tracking controller for redundant robot manipulators. The adaptive controller is implemented to estimate unstructured uncertainties and error reconstruction. In He et al. (2018), one Radial Basis Function Neural Network (RBFNN) is used to estimate the unknown dynamics robotic manipulator. Jung and Hsia (2000) proposed two NN control schemes for a non-model-based robot manipulator, which show advantages over feedback error learning. In Zhang et al. (2018), the authors proposed a gravity compensation based on an RBFNN and robustness analysis, and the results were compared with a classic PID and PD with fixed gravity compensation. Gandolfo et al. (2019) propose a control scheme that combines a classical PD and a robust adaptive compensator based on NNs.

Although adaptive controllers are addressed for systems with non-linear and uncertainties dynamics, thus their slow convergence can lead to performance degradation or even affect operational safety. In Liu et al. (2019), an adaptive NN control with optimal number of hidden nodes and less computation is formulated for approximating the trajectory of robot manipulator. Similarly, Yang et al. (2018) develop a control and identification scheme in order to identify the unknown robot parameters with an enhanced convergence rate. Another approach is to relax the linear parameterized assumption and the requirements of system knowledge, thus, NNs have been used as function approximators. In time series modeling, RBFNN is commonly used for function approximation, since its value is different from zero in infinite space, and its approximation can avoid the local minimum (Wang et al., 2019a). An RBFNN uses a Gauss function as its activation function. In general, RBFNN controllers waste less computational resources in comparison to other NN controllers (He et al., 2018). In Wang et al. (2012), the authors proposed an RBFNN to compensate for non-linear dynamics of the robotic manipulator and a robust control designed to suppress the modeling error of NN.

However, update laws commonly increase the weight magnitudes until the output error has been mitigated, without a robust design continued training can lead to excessive control effort. In order to avoid this, adaptive controls frequently update the neural weights according to robust adaptive laws, which are computed with Lyapunov methods (Razmi and Macnab, 2020). In this work, a robust adaptive control design to compensate a nominal controller for robot manipulator with uncertainties and external perturbations is formulated. Moreover, a scheme based on two RBFNN in cascade is proposed in order to improve the response of the nominal controller. In the scheme aforementioned, the first NN is used to estimate the error and the second uses the estimation error value to improve the output of the nominal controller. NN weights are online updated by developing new adaptive laws. The adaptive law based on the gradient is modified by introducing a recompense function of the online error in order to improve the convergence of the NN weights.

In this work, a PD control with a scheme based on NNs in cascade is designed to manage the compensation of uncertainties in a robot manipulator. The main contributions of this paper are summarized as follows:

- In order to improve the robustness of the system against external disturbance, and unknown system parameters, a scheme of cascade NNs is proposed.
- A recompense function for neural weights updates is proposed in order to improve the NNs' weights convergence.
- The response of the nominal controller is improved.

To validate the proposed scheme and compare it with conventional neural compensation, a two-degree-of-freedom robot manipulator (TDOFRM) is proposed. This paper is organized as follows: Section 2 presents the preliminary mathematical model of the TDOFRM, the conventional PD compensation with RBFNN. Section 3 describes the controller design based on NNs in cascade. Section 4 presents simulation experiments and compared with the conventional compensation, and finally, section 5 presents the conclusions.

## 2. Adaptive Control to Robotic Manipulator

### 2.1. Dynamic Model of Robotic Manipulator

The dynamic model of an *n* degree of freedom robot manipulator can be described as follows (Spong and Vidyasagar, 1989):

(1) $$\begin{array}{l} M\left(q\right)\ddot{q}+C\left(q,\dot{q}\right)\dot{q}+g\left(q\right)=\tau +d \end{array}$$

*M*(*q*) is a *n* × *n* inertia matrix, $C\left(q,\dot{q}\right)$ is a *n* × *n* is a centrifugal and Coriolis matrix, and *g*(*q*) is a *n* × 1 vector of gravity. $q,\dot{q},\ddot{q}$ are the position, velocity, and acceleration of each link, respectively. τ ∈ *R*^*n*^ is the control input and *d* denotes disturbances.

### 2.2. Proportional Derivative Control Scheme

In industrial application, the exact model is difficult to obtain and external disturbances are always present in practice. According to Liu (2013), a nominal model of robot manipulator can be computed as *M*_0_(*q*), $C_{0}\left(q,\dot{q}\right)$, and *g*_0_(*q*). Considering Δ*M* = *M*_0_ − *M*, Δ*C* = *C*_0_ − *C*, and Δ*g* = *g*_0_ − *g*, Equation (1) is reordered as follows:

(2) $$\begin{array}{l} \left(M_{0}\left(q\right)-\Delta M\right)\ddot{q}+\left(C_{0}\left(q,\dot{q}\right)-\Delta C\right)\dot{q}+\left(g_{0}\left(q\right)-\Delta g\right)=\tau +d \end{array}$$

Thus,

(3) $$\begin{array}{l} M_{0}\left(q\right)\ddot{q}+C_{0}\left(q,\dot{q}\right)\dot{q}+g_{0}\left(q\right)=\tau +\Delta M\ddot{q}+\Delta C\dot{q}+\Delta g+d \end{array}$$

Defining $f\left(\cdot \right)=\Delta M\ddot{q}+\Delta C\dot{q}+\Delta g+d$ and if *f* (·) is known, the control law is designed as

(4) $$\begin{array}{l} M_{0}\left(q\right)\left(\ddot{q_{d}}-\kappa_{v}\dot{e}-\kappa_{p}e\right)+C_{0}\left(q,\dot{q}\right)\dot{q}+g_{0}\left(q\right)-f\left(\cdot \right)=\tau \end{array}$$

Submitting Equation (4) into Equation (3), the close loop system can be expressed as follows:

(5) $$\begin{array}{l} \ddot{e}+\kappa_{v}\dot{e}+\kappa_{p}e=0 \end{array}$$

where *e* = *q* − *q*_*d*_, $\dot{e}=\dot{q}-{\dot{q}}_{d}$, and $\ddot{e}=\ddot{q}-{\ddot{q}}_{d}$. Frequently, *f* (·) in industrial applications is unknown, hence, *f* (·) requires to be estimated and compensated.

### 2.3. Radial Basis Function Neural Network Approximation

The NNs approximates *M*(*q*), *C*(*q*), and *g*(*q*) when they are unknown. The Radial Basis Function (RBF) algorithm can approximate a continuous function and it is defined as

(6) $$\begin{array}{l} \phi_{i}=exp^{\left(-\frac{∥x-c_{i}{∥}^{2}}{b_{i}^{2}}\right)},i=1,2,\ldots ,n \end{array}$$

(7) $$\begin{array}{l} y=W\phi \end{array}$$

where *x* is the input vector, ϕ = [ϕ_1_, ϕ_2_, …, ϕ_*n*_] is the output of the Gaussian function, *y* is the output of the NN, *W* is the weight values matrix, and *c*_*i*_ is the center and *b*_*i*_ is the width of the Gaussian function. In Gandolfo et al. (2019); Liu et al. (2019), it has been shown that an RBFNN can approximate a non-linear function *f* (·) under the following assumptions

1. The output $\hat{f}\left(x,W^{*}\right)$ is a continuous function.
2. Given a small positive constant ϵ_0_ and a continuous function *f* (·), a weight vector W^*^ exists so that $\hat{f}\left(\cdot \right)$ satisfies.
  (8) $$\begin{array}{l} max∥f\left(\cdot \right)-{\hat{f}}^{*}\left(\cdot \right)∥\leq \varepsilon_{0} \end{array}$$
  and $W^{*}=\underset{W\in \beta \left(M_{W}\right)}{\text{argmin}}\left\{\underset{W\in \beta \left(M_{W}\right)}{\text{sup}}∥f\left(\cdot \right)-{\hat{f}}^{*}\left(\cdot \right)∥\right\}$, where W^*^ is *n* × *n* matrix that denotes the optimal weigh values for *f* (·) approximation.

### 2.4. Adaptive Law to Compensation Control

In the controller scheme proposed (Feng, 1995), the close loop system is given by the following equation:

(9) $$\begin{array}{l} \tau =M_{0}\left(q\right)\left(\ddot{q_{d}}-\kappa_{v}\dot{e}-\kappa_{p}e\right)+C_{0}\left(q,\dot{q}\right)\dot{q}+g_{0}\left(q\right)-\hat{f}\left(\cdot \right) \end{array}$$

where $\hat{f}\left(\cdot \right)=\hat{W}\phi \left(x\right)$ and $\hat{W}$ is an estimation of W^*^. Equations (1) and (9) have the same term, and the substitution result is shown in the following equation:

(10) $$\begin{array}{l} M\left(q\right)\ddot{q}+C\left(q,\dot{q}\right)\dot{q}+g\left(q\right)-d\\ \begin{array}{l} \;=M_{0}\left(q\right)\left(\ddot{q_{d}}-\kappa_{v}\dot{e}-\kappa_{p}e\right)+C_{0}\left(q,\dot{q}\right)\dot{q}+g_{0}\left(q\right)-\hat{f}\left(\cdot \right) \end{array} \end{array}$$

Then, the equation $M_{0}\left(q\right)\ddot{q}+C_{0}\left(q,\dot{q}\right)\dot{q}+g_{0}\left(q\right)$ is subtracted with Equation (10) in both sides as follows:

(11) $$\begin{array}{l} M_{0}(q)\ddot{q}+C_{0}(q,\dot{q})\dot{q}+g_{0}(q)-\left[M(q)\ddot{q}+C(q,\dot{q})\dot{q}+g(q)-d\right]\\ \;=M_{0}(q)\ddot{q}+C_{0}(q,\dot{q})\dot{q}+g_{0}(q)-[M_{0}(q)(\overset{..}{q_{d}}-\kappa_{v}\dot{e}-\kappa_{p}e)\\ \;+C_{0}(q,\dot{q})\dot{q}+g_{0}(q)-\hat{f}(\cdot )] \end{array}$$

The result is given as follows:

(12) $$\begin{array}{l} M_{0}^{-1}\left(q\right)\left[\Delta M\ddot{q}+\Delta C\dot{q}+\Delta g\left(q\right)+d\right]\\ \begin{array}{l} \;=\ddot{e}+\kappa_{v}\dot{e}+\kappa_{p}e+M_{0}^{-1}\left(q\right)\left[\hat{f}\left(\cdot \right)\right] \end{array} \end{array}$$

Equation (12) can be rewritten as follows:

(13) $$\begin{array}{l} \ddot{e}+\kappa_{v}\dot{e}+\kappa_{p}e=M_{0}^{-1}\left(q\right)\left[f\left(\cdot \right)-\hat{f}\left(\cdot \right)\right] \end{array}$$

Select to *x* = (*e* $\dot{e}$)^*T*^. Equation (13) turns into

(14) $$\begin{array}{l} \dot{x}=Ax+B\left(f\left(\cdot \right)-\hat{f}\left(\cdot \right)\right) \end{array}$$

where

(15) $$\begin{array}{l} A=\left(\begin{array}{cc} 0 & I\\ -\kappa_{p} & -\kappa_{v} \end{array}\right);B=\left(\begin{array}{c} 0\\ M_{0}^{-1}\left(q\right) \end{array}\right) \end{array}$$

Setting $f\left(\cdot \right)-\hat{f}\left(\cdot \right)=f\left(\cdot \right)-{\hat{f}}^{*}\left(\cdot \right)+{\hat{f}}^{*}\left(\cdot \right)-\hat{f}\left(\cdot \right)=\zeta +W^{*T}\phi -{\hat{W}}^{T}\phi =\zeta -{\tilde{W}}^{T}\phi$ and $\tilde{W}=\hat{W}-W^{*},\zeta =f\left(\cdot \right)-{\hat{f}}^{*}\left(\cdot \right)$, where ζ denotes the modeling error due to the use of the NN. The modeling error ζ is bounded by a finite constant ζ_0_, where $\zeta_{0}=\underset{t\geq 0}{\text{sup}}∥f\left(\cdot \right)-{\hat{f}}^{*}\left(\cdot \right)∥$. Finally,

(16) $$\begin{array}{l} \dot{x}=Ax+B\left(\zeta -{\tilde{W}}^{T}\phi \right) \end{array}$$

The Lyapunov function is given by the following equation:

(17) $$\begin{array}{l} L=\frac{1}{2}x^{T}Px+\frac{1}{2\varphi }∥\tilde{W}{∥}^{2};\varphi >0 \end{array}$$

where $\tilde{W}=\hat{W}-W^{*}$ is a definition that describes the estimation error. In Equation (17), *P* is a positive definite matrix that satisfies the Lyapunov equation

(18) $$\begin{array}{l} PA+A^{T}P=-Q;Q\geq 0 \end{array}$$

Equation (17) can be rewritten in terms of the next definition

(19) $$\begin{array}{l} ∥\tilde{W}{∥}^{2}=\underset{i,j}{\sum }|w_{ij}{|}^{2}=tr\left(\tilde{W}{\tilde{W}}^{T}\right)=tr\left({\tilde{W}}^{T}\tilde{W}\right) \end{array}$$

Thus, the derivative of *V* is given as follows:

(20) $$\begin{array}{l} \dot{L}=\frac{1}{2}\left[x^{T}P\dot{x}+{\dot{x}}^{T}Px\right]+\frac{1}{\varphi }tr\left({\dot{\tilde{W}}}^{T}\tilde{W}\right) \end{array}$$

Substituting Equation (16) for (20), the results is given by

(21) $$\begin{array}{l} \dot{L}=\frac{1}{2}[x^{T}P(Ax+B(\zeta -{\tilde{W}}^{T}\phi ))\\ \;+{\left(Ax+B\left(\zeta -{\tilde{W}}^{T}\phi \right)\right)}^{T}Px]+\frac{1}{\varphi }tr({\dot{\tilde{W}}}^{T}\tilde{W})\\ \;=\frac{1}{2}[x^{T}PAx+x^{T}PB(\zeta -{\tilde{W}}^{T}\phi )+x^{T}A^{T}Px)\\ \;+{\left(\zeta -{\tilde{W}}^{T}\phi \right)}^{T}B^{T}Px]+\frac{1}{\varphi }tr({\dot{\tilde{W}}}^{T}\tilde{W})\\ \;=\frac{1}{2}[x^{T}PAx+x^{T}A^{T}Px+x^{T}PB(\zeta -{\tilde{W}}^{T}\phi ))\\ \;+{\left(\zeta -{\tilde{W}}^{T}\phi \right)}^{T}B^{T}Px]+\frac{1}{\varphi }tr({\dot{\tilde{W}}}^{T}\tilde{W})\\ \;=\frac{1}{2}[x^{T}(PA+A^{T}P)x+x^{T}PB\zeta -x^{T}PB{\tilde{W}}^{T}\phi )\\ \;+\zeta^{T}B^{T}Px-\phi^{T}\tilde{W}B^{T}Px]+\frac{1}{\varphi }tr({\dot{\tilde{W}}}^{T}\tilde{W})\\ \;=-\frac{1}{2}[x^{T}Qx]+\zeta^{T}B^{T}Px-B^{T}\tilde{W}\phi^{T}Px+\frac{1}{\varphi }tr({\dot{\tilde{W}}}^{T}\tilde{W}) \end{array}$$

Considering that *x*^*T*^*PBζ* = ζ^*T*^*B*^*T*^*Px*, $x^{T}PB{\tilde{W}}^{T}\phi =\phi^{T}\tilde{W}B^{T}Px$ and $\phi^{T}\tilde{W}B^{T}Px=tr\left[B^{T}Px\phi^{T}\tilde{W}\right]$ in Equation (21), this results in

(22) $$\begin{array}{l} \dot{L}=-\frac{1}{2}\left[x^{T}Qx\right]+\zeta^{T}B^{T}Px+\frac{1}{\varphi }tr\left(-\varphi B^{T}Px\phi^{T}\tilde{W}+{\dot{\tilde{W}}}^{T}\tilde{W}\right) \end{array}$$

### 2.5. Adaptive Control Based on Cascade Neural Network

**Figure 1:**
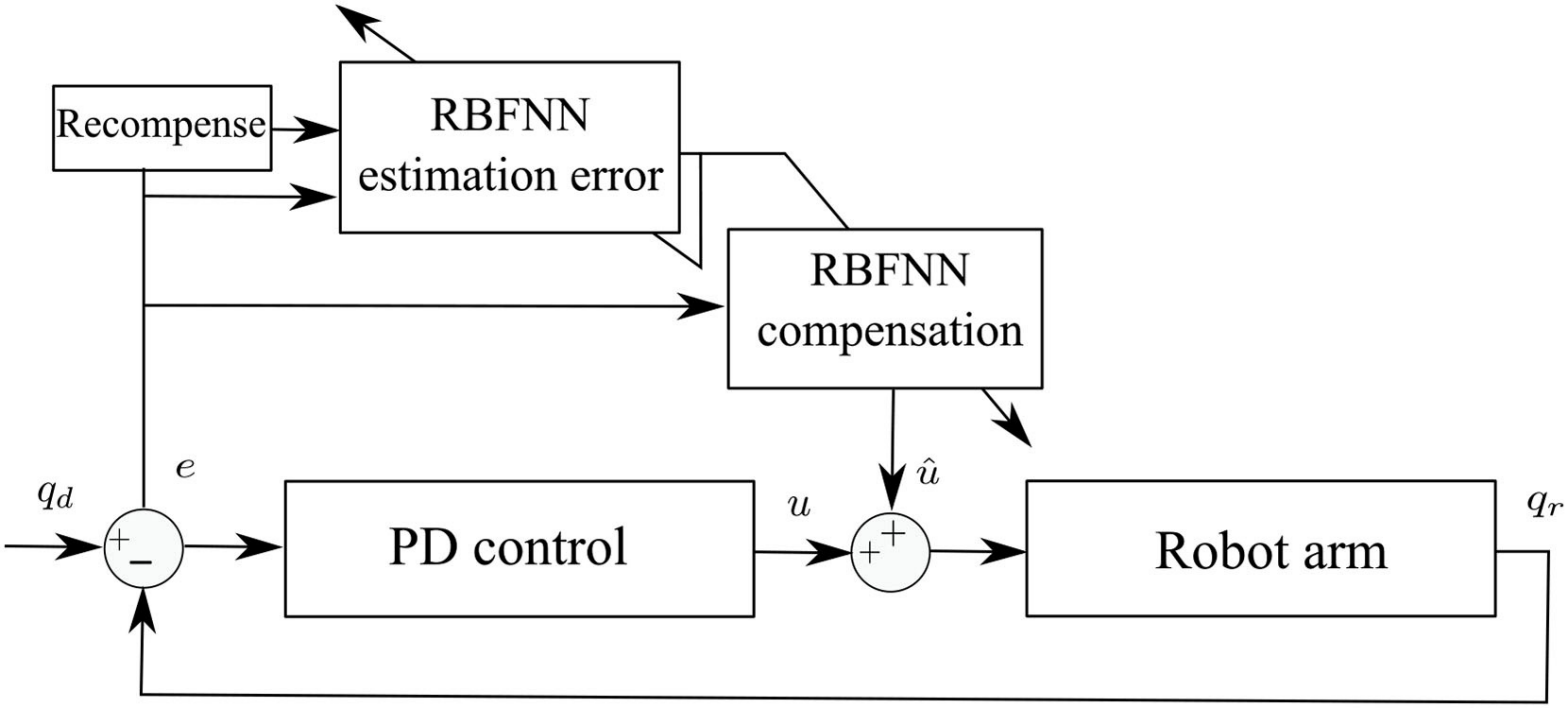
Proposed adaptive control scheme.

The PD control has been widely implemented in robot control to deal with the drawbacks presented by the integer gain in PID control. However, the PD control can have similar deficiencies if the derivative gain has high values (Wen Yu and Rosen, 2013). The PD control with compensation presents positive results to avoid high derivative gains and identify uncertainties that occur in the real operation of robot manipulators. In this work, a compensation of the nominal controller is proposed. The adaptive control scheme is shown in Figure 1. Two RBFNN in cascade are proposed to deal with the tracking error and the NN weight estimation error.

#### 2.5.1. NN Weight Estimation Error

The input of NN is an error vector defined as follows:

(23) $$\begin{array}{l} x=\left[\begin{array}{c} e\\ \dot{e} \end{array}\right]=\left[\begin{array}{c} q_{i}-q_{\vartheta i}\\ {\dot{q}}_{i}-{\dot{q}}_{\vartheta i} \end{array}\right] \end{array}$$

where *i* and ϑ denote the real and desired position and velocity of the n-link of the robot. The NN estimation error anticipates which action to take in order to improve the output of the nominal control. Thus, the prediction of estimation error is also given as the output of RBFNN as follows:

(24) $$\begin{array}{l} \hat{x}=\overset{J}{\underset{j=1}{\sum }}W_{e}\phi_{e} \end{array}$$

where ϕ_*e*_ is the Gaussian function given by Equation (6). The criterion for the weight update is proposed in the following equation:

(25) $$\begin{array}{l} {\dot{W}}_{e}=\gamma_{e}r\phi_{e}{\left(x\right)}^{T}PB \end{array}$$

where $r=∥e^{-x_{e}^{2}}∥$ is a function that represents the recompense signal. The goal of the recompense function is to lead longer traces for an identification problem.

#### 2.5.2. Adaptive Law to Compensate the Nominal Controller

A novel adaptive control scheme is proposed to ensure that the output of the nominal controller for the system defined in Equation (1) reaches the position desired, and the estimated NN can converge an ideal weight. An RBFNN is selected to approximate the system dynamics and deals with uncertainties and external disturbances, which is given as follows:

(26) $$\begin{array}{l} {\hat{f}}^{*}=\overset{J}{\underset{j=1}{\sum }}\hat{W}\phi_{c} \end{array}$$

where ϕ_*c*_ is a Gaussian function given by the equation form 6. The NN actor provides compensation to the PD controller to, that is, $\hat{f}\left(\cdot \right)={\hat{f}}^{*}$. The weigh update of the NN actor is proposed according to the following equation:

(27) $$\begin{array}{l} \dot{\hat{W}}=\gamma_{c}\phi_{c}{\left(\hat{x}+\left(\hat{x}-x\right)\right)}^{T}PB \end{array}$$

If we select the parameter update law as $\dot{\tilde{W}}=\dot{\hat{W}}$, we assume that the value $x\approx \hat{x}+\left(\hat{x}-x\right)$. Substituting Equation (27) into Equation (22), the result is given as

(28) $$\begin{array}{l} \dot{L}=-\frac{1}{2}\left[x^{T}Qx\right]+\zeta^{T}B^{T}Px \end{array}$$

As it is well-known, $∥\zeta^{T}∥\leq ∥\zeta_{0}∥$ and from Equation (15), $∥B∥=∥M_{0}^{-1}\left(q\right)∥$, and Equation (28) can be written as follows:

(29) $$\begin{array}{l} \dot{L}\leq -\frac{1}{2}\lambda_{min}\left(Q\right)∥x{∥}^{2}+∥\zeta_{0}∥∥M_{0}^{-1}\left(q\right)∥\lambda_{max}\left(P\right)∥x∥ \end{array}$$

(30) $$\begin{array}{l} \dot{L}=-\frac{1}{2}∥x∥\left[\lambda_{min}\left(Q\right)∥x∥-2∥\zeta_{0}∥∥M_{0}^{-1}\left(q\right)∥\lambda_{max}\left(P\right)\right] \end{array}$$

where λ_*min*_ denotes the minimum eigenvalues of matrix Q and λ_*max*_ denotes the maximum eigenvalues of matrix P. In order to satisfy $\dot{L}\leq 0$, the value of ∥*x*∥ should be satisfied as follows:

(31) $$\begin{array}{l} ∥x∥\leq \frac{2∥M_{0}^{-1}\left(q\right)∥\lambda_{max}\left(P\right)}{\lambda_{min}Q}∥\zeta_{0}∥ \end{array}$$

According to Equation (28), $\dot{L}$ is negative semidefinite, that is *L*(*x, W, t*) ≤ *L*(*x, W*, 0). It implies that *x*, and *W* are bounded. Let function $\Omega =\dot{L}$ and integrate Ω with respect to time as follows:

(32) $$\begin{array}{l} \int_{0}^{t}\Omega \left(s\right)ds\leq L\left(x,W,t\right)\leq L\left(x,W,0\right) \end{array}$$

Due to *L*(*x, W*, 0) is bounded, and *L*(*x, W, t*) is non-increasing and bounded, the following result can be computed:

(33) $$\begin{array}{l} \underset{t\to \infty }{\text{lim}}\int_{0}^{t}\Omega \left(s\right)ds<\infty \end{array}$$

Since $\dot{\Omega }\left(s\right)$ is bounded, by Barbalat's lemma (Slotine and Li, 1991), ${\text{lim}}_{t\to \infty }\Omega \left(s\right)=0$, that is *x* → 0 as *t* → ∞.

## 3. Results

**Figure 2:**
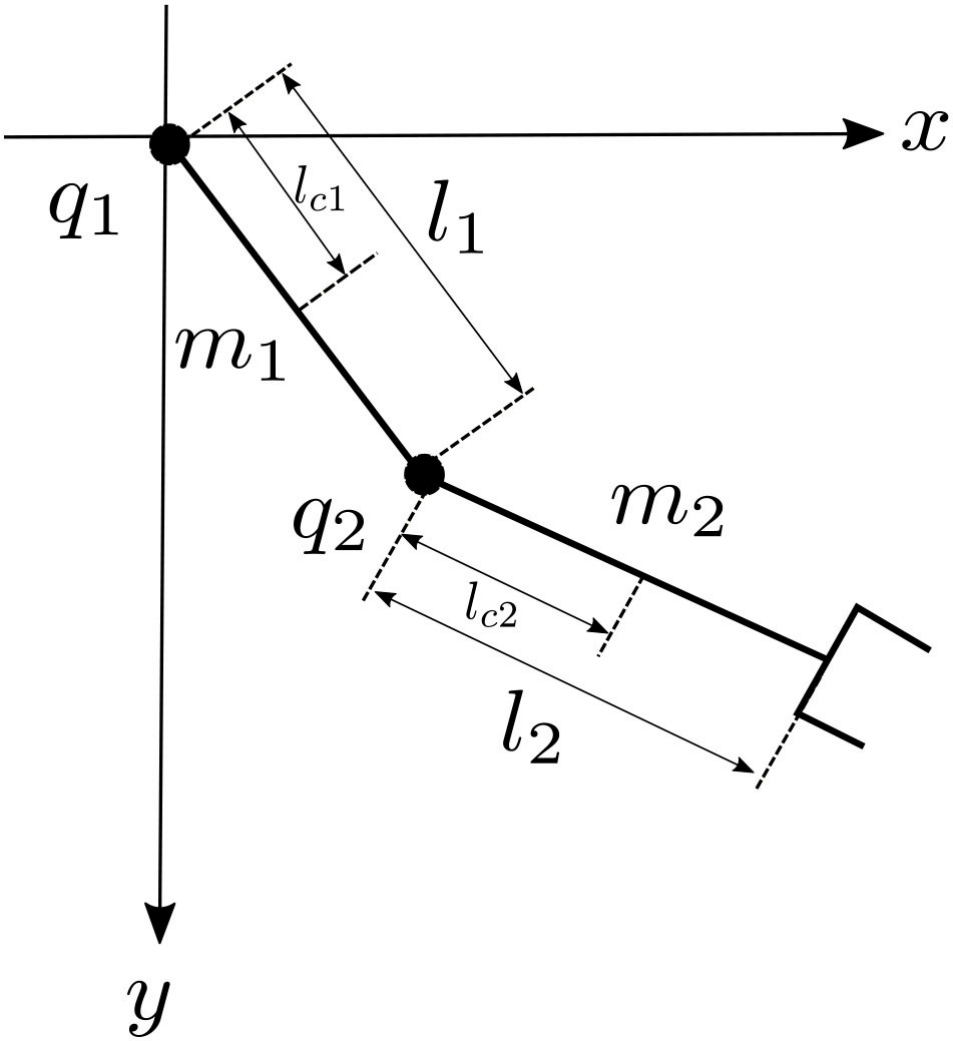
Model of two degree-of-freedom (DOF) robot manipulator.

In order to validate the proposed scheme of control, a set of simulations was carried out. Two simulations are proposed, the first considers an adaptive control using an RBFNN, and the second is based on the proposed scheme using two RBFNNs in cascade. The controllers were also implemented in a TDOFRM, which is shown in Figure 2. The main objective of controllers is the position tracking in the presence of external disturbances.

(34) $$\begin{array}{l} \left[\begin{array}{c} \tau_{1}\\ \tau_{2} \end{array}\right]=\left[\begin{array}{cc} M_{11}\left(q\right) & M_{12}\left(q\right)\\ M_{21}\left(q\right) & M_{22}\left(q\right) \end{array}\right]\left[\begin{array}{c} {\ddot{q}}_{1}\\ {\ddot{q}}_{2} \end{array}\right]\\ \begin{array}{l} \;+\left[\begin{array}{cc} C_{11}\left(q,\dot{q}\right) & C_{12}\left(q,\dot{q}\right)\\ C_{21}\left(q,\dot{q}\right) & C_{22}\left(q,\dot{q}\right) \end{array}\right]\left[\begin{array}{c} {\dot{q}}_{1}\\ {\dot{q}}_{2} \end{array}\right]+\left[\begin{array}{c} g_{1}\left(\dot{q}\right)\\ g_{2}\left(\dot{q}\right) \end{array}\right] \end{array} \end{array}$$

where:

$$\begin{array}{l} \;M_{11}\left(q\right)=m_{1}l_{c1}^{2}+m_{2}l_{1}^{2}+m_{2}l_{c2}^{2}+2m_{2}l_{1}l_{c2}cos\left(q_{2}\right)+I_{1}+I_{2}\\ \;M_{12}\left(q\right)=m_{2}l_{c2}^{2}+m_{2}l_{1}l_{c2}cos\left(q_{2}\right)+I_{2}\\ \;M_{21}\left(q\right)=m_{2}l_{c2}^{2}+m_{2}l_{1}l_{c2}cos\left(q_{2}\right)+I_{2}\\ \;M_{22}\left(q\right)=m_{2}l_{c2}^{2}+I_{2}\\ \;C_{11}\left(q,\dot{q}\right)=-m_{2}l_{1}l_{c2}sin\left(q_{2}\right){\dot{q}}_{2}\\ \;C_{12}\left(q,\dot{q}\right)=m_{2}l_{1}l_{c2}sin\left(q_{2}\right)\left({\dot{q}}_{1}+{\dot{q}}_{2}\right)\\ \;C_{21}\left(q,\dot{q}\right)=m_{2}l_{1}l_{c2}sin\left(q_{2}\right){\dot{q}}_{1}\\ \;C_{22}\left(q,\dot{q}\right)=0\\ \;g_{1}\left(q\right)=\left(m_{1}l_{c1}+m_{2}l_{1}\right)gsin\left(q_{1}\right)+m_{2}l_{c2}gsin\left(q_{1}+q_{2}\right)\\ \begin{array}{l} \;g_{2}\left(q\right)=m_{2}l_{c2}gsin\left(q_{1}+q_{2}\right) \end{array} \end{array}$$

The parameters came from Kelly and Santibáñez (2003) and presented in Table 1.

**Table 1:** Robot manipulator parameters.

**Parameter**	**Value**	**Units**
*l*_1_	0.45	*m*
*l*_2_	0.45	*m*
*l*_*c*1_	0.091	*m*
*l*_*c*2_	0.048	*m*
*m*_1_	23.902	*kg*
*m*_2_	3.880	*kg*
*I*_1_	1.266	*kgm*^2^
*I*_1_	0.093	*kgm*^2^
*g*	9.81	$\frac{m}{s^{2}}$

The desired position vector is defined as follows:

(36) $$\begin{array}{l} q_{d}=\left[\begin{array}{c} q_{1d}\\ q_{2d} \end{array}\right]=\left[\begin{array}{c} \frac{\pi }{6}sin\left(\frac{1}{4}\pi t\right)\\ \frac{\pi }{6}sin\left(\frac{2}{3}\pi t\right) \end{array}\right] \end{array}$$

The initial positions are given by $q_{0}={\left[\begin{array}{cc} \frac{\pi }{12} & \frac{\pi }{12} \end{array}\right]}^{T}$. The uncertainties and unknown disturbances are defined as follows:

(37) $$\begin{array}{l} d=\left[\begin{array}{c} sin\left(2\pi t\right)+cos\left(\frac{1}{4}\pi t\right)\\ sin\left(2\pi t\right)+cos\left(\frac{1}{4}\pi t\right) \end{array}\right] \end{array}$$

The matrix *Q*, *A*, and *B* are

(38) $$\begin{array}{l} \;Q=\left[\begin{array}{cccc} 50 & 0 & 0 & 0\\ 0 & 50 & 0 & 0\\ 0 & 0 & 50 & 0\\ 0 & 0 & 0 & 50 \end{array}\right];\\ \begin{array}{lll} A & = & \left[\begin{array}{cccc} 0 & 0 & 1 & 0\\ 0 & 0 & 0 & 1\\ -0.25 & 0 & -1 & 0\\ 0 & -0.25 & 0 & -1 \end{array}\right];B=\left[\begin{array}{cc} 0 & 0\\ 0 & 0\\ 1 & 0\\ 0 & 1 \end{array}\right] \end{array} \end{array}$$

Two RBFNNs are proposed for critic and actor agents. Here, *γ*_*e*_ is the learning rate of the RBFNN estimation error and *γ*_*c*_ is the learning rate of the RBFNN tracking error; *c*_*i*_ is the center vector of neural net *i* and *b* is the width value of Gaussian function for neural net *i*. The next values are proposed due to the optimal weight for the actor NN and critic NN could take arbitrary large values, but in order to avoid any numerical problems, this work considers *γ*_*e*_ = 0.5, *γ*_*c*_ = 0.4, *c*_*i*_ = $\left[\begin{array}{ccccc} -2 & -1 & 0 & 1 & 2 \end{array}\right]$, *b*_*i*_ = 0.5, and *i* = 4.

**Figure 3:**
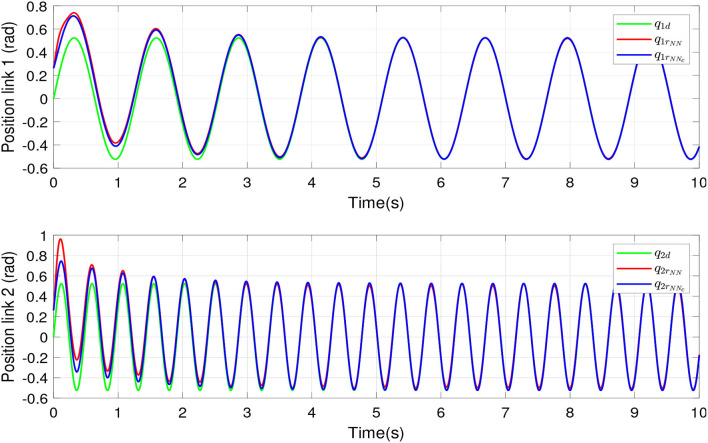
Real and desired links positions of the robot manipulator.

Figure 3 shows the tracking position of links 1 and 2, where *NN* and *NN*_*c*_ indicate an RBFNN compensation and a compensation based on two RBFNN in cascade, respectively. The green lines show the desired tracking position. The red lines indicate the tracking position of NN compensation. The blue lines show the tracking position of the proposed controller. The uncertainties and disturbances were added to the controller. According to the RBFNN in cascade, an RBFNN predicts the NN estimation error, and this value is included in adaptive laws to update the RBFNN compensation in order to take adequate action for the disturbances and guarantee the convergence of tracking error. The proposed recompense function helps to maintain longer traces for the identification task over time.

**Figure 4:**
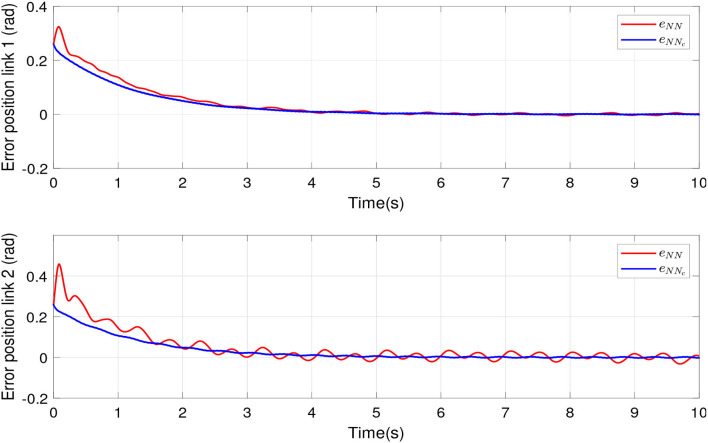
Tracking error of the robot manipulator joints by scheme proposed and conventional compensation.

The compensation of proposed algorithm is compared with an RBFNN compensation. Figure 4 shows tracking errors for links 1 and 2. The red line represents the tracking error of an RBFNN for compensation, which presents overshoot to reach the desired positions and oscillations in steady state. The blue line indicates the tracking error of the proposed algorithm, which present robustness against uncertainties and disturbances. The green line indicates the desired tracking positions for links 1 and 2.

**Figure 5:**
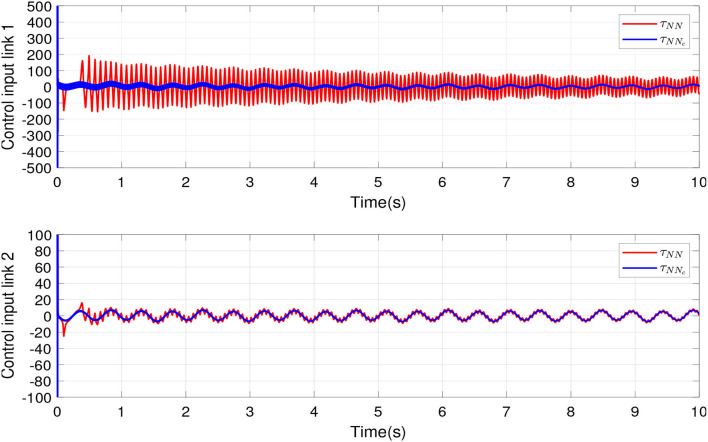
Control inputs of links 1 and 2.

The simulations were proposed in order to show the difference between adaptive conventional control and the proposed scheme. In this sense, two desired tracking signal was proposed that goes at different velocities, and link 1 follows a slow signal and link 2 follows a fast signal. Figure 5 shows the control inputs to links 1 and 2, and it also exhibits the improvement of the nominal controller under our scheme proposed in comparison with adaptive conventional control.

**Figure 6:**
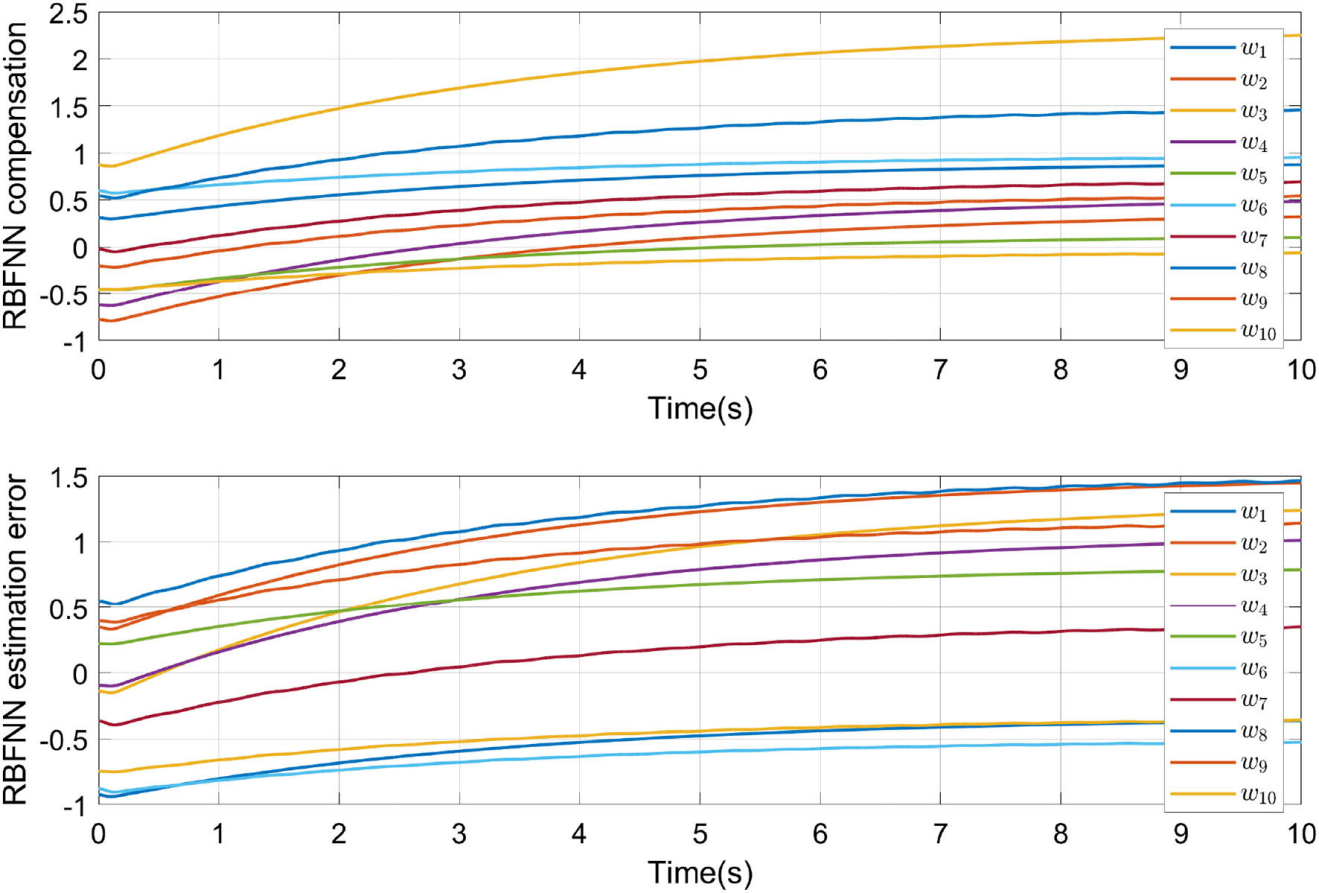
Convergence of neural networks parameters.

In Figure 6, the NN weights convergence process of the two RBFNN in cascade are shown. Figure 6 also denoted as the identification process in order to deal with the uncertainties and external disturbances is reached.

The important factors that usually must be considered together are time and error. A performance index is a measure that indicates those features of the response that are regarded to be important. In order to evaluate the performance of the proposed controller, a comparison of different performance indices is shown in Table 2. Hence, Table 2 is based on the next four equations, integral absolute error (IAE), integral square error (ISE), integral time absolute error (ITAE), and integral time square error (ITSE).

(39) $$\begin{array}{l} ISE=\int e{\left(t\right)}^{2}dt \end{array}$$

(40) $$\begin{array}{l} IAE=\int |e\left(t\right)|dt \end{array}$$

(41) $$\begin{array}{l} ITSE=\int t\;e{\left(t\right)}^{2}dt \end{array}$$

(42) $$\begin{array}{l} ITAE=\int t|e\left(t\right)|dt \end{array}$$

Table 2 shows that the proposed controller presents a better response than the conventional PD control compensation based on an RBFNN. According to the performance indices of links 1 and 2, the proposed scheme presents an adequate response against external disturbance. Moreover, it presents less oscillation in steady state and less time in the transient response than the conventional compensation based on an RBFNN.

**Table 2:** Comparison of different errors, ITAE, ITSE, IAE, and ISE as performance indices.

**Controller**	**Indices**	**Link 1**	**Link 2**
PD+NN	IAE	0.3758	0.4992
	ISE	0.0554	0.0869
	ITAE	0.5162	1.0030
	ITSE	0.0340	0.0578
PD+NN_C_	IAE	0.3057	0.3077
	ISE	0.0373	0.0370
	ITAE	0.3898	0.4148
	ITSE	0.0232	0.0231

## 4. Conclusions

The algorithm proposed has been implemented to compensate for the PD control of a TDOFRM. A PD control was selected because the common knowledge that if designed with gravity compensation, it can reach asymptotic stability. The cascade scheme was implemented by two RBFNN in cascade, which compensates for the control input in order to deal with the estimation error, uncertainties, and external disturbances. The proposed algorithm was validated using a simulation of a TDOFRM. Two adaptive algorithms for compensating for the controller of robot manipulators were implemented. The first was based on a conventional RBFNN, and the second is the proposed algorithm that uses two RBFNN. An adaptive law is proposed to deal with the simultaneous convergence of both NNs, which are used to estimate for tracking error and estimation error. The results showed that the proposed compensation scheme based on RBFNN presents robustness against external uncertainties and disturbances, also an adequate convergence for the NN weights. Position tracking has been reached without overshoots and oscillations in steady state in comparison to compensations with a conventional RBFNN scheme.

## Data Availability Statement

The raw data supporting the conclusions of this article will be made available by the authors, without undue reservation.

## Author Contributions

JB and GH proposed a two-degree-of freedom robot manipulator to validate the scheme proposed and they also design the robot manipulator simulation. JV-N and DB contributed to the analysis and discussion of results. LS and EZ contributed with the theoretical analysis, simulations, and also written the main text. All authors participated and contributed to the final version manuscript.

## Conflict of Interest

The authors declare that the research was conducted in the absence of any commercial or financial relationships that could be construed as a potential conflict of interest.
